# Artifacting Identity. How *Grillz*, Ball Gags and Gas Masks Expand the Face

**DOI:** 10.1007/s11245-022-09819-9

**Published:** 2022-09-07

**Authors:** Cristina Voto, Elsa Soro

**Affiliations:** ERC FACETS Post-Doctoral Fellow, Department of Philosophy and Educational Sciences, University of Turin, Via Sant’Ottavio 20, 10124 Turin, Italy

**Keywords:** Agency, Artifacts, Embodiment, Face studies, Identity, Mouth

## Abstract

By questioning the attribution of a primary role to the eyes as bearers of identity within traditional Western culture, this paper will problematize the agentivity performed by the lower mereology of the face, identified with the mouth-nose assemblage. In particular, the study will focus on the manipulation of such facial spatiality through the intervention of three “lower face” artifacts: the grill, the ball gag and the gas mask. This piece of work will examine their plastic and figurative dimensions in the technological interaction with the facial organs. Furthermore, we will take into consideration the sociocultural context of wearability performed by the different bearers with the aim of grasping the identity shift that the artifacts trigger. The study, therefore, will organize the corpus as a sequence that starts inside the oral cavity where the grill is worn; then moves to a progressive exteriority with the ball gag that emerges from the mouth through the straps fastened around the head; eventually dealing with the exterior projection operated by the gas mask which by means of its filters portends beyond the anatomical face. Ultimately the three artifacts are presented as a threefold articulation of a liminal agency towards an expanded form of humanity including animality embedded within and without the space of meaning represented by the face.

## Prologue: The Eyes Without a Face

In 1984 English punk-rock singer Billy Idol released, as the second single from his album *Rebel Yell*, the song *Eyes without a face*. Soundly characterized by synth strings and percussion handclaps, this electro-romantic ballad inspires us to introduce the core of our investigation. In Billy Idol’s song, a chant on lost love and the pain of remembrance, the refrain goes like this: “your eyes without a face got no human grace”. The memory of the eyes of the beloved subtracted from the quintessentially identifying device of the human body, the face (Beling 2017; Ekman 1978; Leone 2019a, b; Marino 2021a, b; Soro 2021; Voto 2020), affects the aesthetic aspect of reminiscences of the lost lover’s very identity. Later, the verse is echoed in the outro with the use of anastrophe and alliteration that intensify the scope of the lament: “such a human waste your eyes without a face”. The song reiterates the title in its refrain, arranging it in a verse that we will use to frame the key issue underpinning our inquiry: in what conditions and through what artifacts is humanity embodied in someone’s face? And once embedded, how does humanity interact with the social performances of identity? To answer this question, throughout these pages, we will deal with *faces without eyes.*

## The Eyes and the Face: An Introduction Beyond the Scopic Dimension of Humanity

The refrain of the Billy Idol song allows us to configure a first proposal suggesting the existence of a web of deep, significant relationships between the agency of the eyes and the quality of being human. There is an established aesthetic and cultural strand according to which the eye is the pivotal element of the face and therefore the indicator of humanity. Furthermore, this ocular-facial correlation can be extended to all the results of the act of looking as the performance of a human gaze, a long-standing tradition of views, sights and perspectives codified not just in visual culture but also within the domains of epistemology (Berger 1972; Crary 1992). To support this proposition, we can trace how in the song the absence of a faciality interacting with the organs of sight is presented as capable of affecting the human nature of the beloved partner. The eyes ripped from the face have consequences not just on the appearance of the lover’s humanity—“they got no human grace”—but the repercussions transcend matter itself to the point of their losing all value—“they are such a human waste”.

In this faciality overwhelmed by the eyes, or maybe it would be better to say, revolving around the ocular organs, many cultural discourses resonate that have traversed human history, which in this circumstance we will inscribe within the Western visual paradigm, from the all-seeing eye of God to the panoptic eye of the Big Brother depicted by George Orwell in his novel *1984*; from the beastly eye of the Cyclops in the *Odyssey* (Farinelli 2003) to the uncanny eye of Hal 9000 (Surace 2021)—the acronym for the *Heuristic ALgorithmic* computer on board the spaceship Discovery in *2001: A Space Odyssey,* a film by Stanley Kubrick (1968) and a science-fiction novel by Arthur Charles Clarke (1968); from the pathologized eye of Alex in *The Clockwork Orange*—the protagonist of the novel by Anthony Burgess (1962) and the film by Stanley Kubrick (1971)—to the iconic eye-cutting in *Le Chien Andalou* by Luis Buñuel (1929), the eyes without a face definitely have not just “got no human grace” but no humanity either.^1^ These ocular remixes and remaking, tokens of a cultural interest in the figurative type of the hyperbolic eye, trace a common expressive pattern that embodies the scopic dimension in the quality of being a human.

This cultural isotopy acts on the face, the device par excellence for the (re)presentation of identity. Let us consider the multiple everyday strategies of identity-making that we all recognize and perform through and within the face: masking, veiling, making up, tattooing, hair-styling, piercing, expression and emotion control, just to mention the most evident and common instances (Magli 2013; Leone 2019a, b). Furthermore, in the current pandemic contemporaneity, everyone has deeply and constantly experimented the impact of covering and face-masking on social communication and interactions (Leone, 2020; Marini, Ansani, Paglieri, Caruana, Viola 2021; Jardim 2022). Moreover, in our hypermediated digital societies, it is difficult not to realize that we are witnessing a new interest in the face and its reproductions which, through the pervading logic of commodification guaranteed by blockchains, requires us to reconsider meanings and phenomenological issues.

The face is a pervasive feature in the communicative design of humanity and perhaps it is precisely its constitutive ubiquity that made it, in the course of history, an elusive object of study, at least until the twentieth century and the development of what Tomas Macho has defined as the *facial society* (1996). In this regard, it is precisely starting from the second half of the past century that a deep-seated philosophical debate began to take shape. In the post-modern era, the face has become the focus of considerable reflection, an entity perceived as being at the same time biological, phenomenological and plastic, an interfacing frontier. A tensive space between identity and otherness, as developed in the pages of Emmanuel Lévinas’ *Totalité et Infini. Essai sur l'extériorité* (1961) which centers on the experience of the Holocaust, through *Mille Plateaux* (1980) by Gilles Deleuze and Félix Guattari where the category of faciality is described as the Western normative abstract machine based on the portrait of Christ or, more recently, the reworkings of Lévinas’ perspective effected by Judith Butler on the reproductions of the faces of victims and perpetrators in *Precarious Life: The Powers of Mourning And Violence* (2004), written in the aftermath of the war on terror declared by George W. Bush. But the face has also been read and analyzed as the interspecies fronter between human animals and non-human animals by philosophers such as Jacques Derrida in *L'animal que donc je suis* (2006) or biologists like Donna Haraway in her essay *When species meet* (2008). The transversal study of these authors constitutes for us the framework of our research.

Starting from this theoretical perspective, we are interested in the visible interactions that occur in the mereology of the face when the eyes are excluded. To frame this phenomenon, we will resort to three artifacts that are embedded in the lower face while embodying an expanded form of humanity including animality. Thanks to this enactment the three artifacts stage forms of life perceived by the self or by others as subversive. But in what sense can we speak about an agentivity of artifacts?

A strong model of intentionality predominates in Western culture, leading us to consider something an *action* only if there is a willing human who aspires to it. Within this horizon acting seems to be characterized as a conscious and voluntary doing, therefore also responsible from a moral, ethical and legal point of view. In this respect, an agent—someone who is committed to doing an act—is a person who determines the effects of the action according to a plan (Volli 2009). As a consequence of this strong anthropomorphic ideologization concerning acting, artifacts are often interpreted as deprived of an acting capacity since their agency is perceived as absorbed by their function. Roland Barthes (1964) was probably the first to recognize this hypertrophy of significance in the artifacts of the world that brought him to investigate the meaning that goes beyond use. The social connotation of artifacts transforms them into signs of their own meaning, that is, converting the use into a sign of use, to the point of presenting them as simple objects, refusing to admit their meaning in the name of a presumed naturalness. But if we are to study the meaning of artifacts, we must assume a detachment guaranteed by a different consideration on what agency, as a capacity to intervene, is and what it can configure.

Regardless of their non-anthropomorphic nature, artifacts are often inclined towards a doing, beyond a moral, ethical or legal—anthropocentric—perspective, an enactment resulting from the interaction between organic and non-organic matters. As Bruno Latour stated: “action must be shared with other kinds of actants dispersed in other spatio-temporal frameworks and who exhibit other kinds of ontology” (Latour 1996: 239). Reworking the notion of agency, though, implies considering relational ontologies. Agency is not possessed, it is not a property of people or artifacts; rather, agency is an actualization, a matter of the possibility of reconfiguring entanglements. In the words of Karen Barad, agency is:

“Not something *that* someone or something *has.* It cannot be designated as an attribute of subjects or objects (as they do not preexist as such). It is not an attribute whatsoever. Agency is “doing” or “being” in its intra-activity. It is the enactment of *iterative changes* to *particular practices-iterative* reconfiguring of *topological* manifolds of *spacetime-matter* relations- through the dynamics of intra-activity. Agency is *about changing* possibilities of change entailed in *reconfiguring material-discursive apparatuses* of bodily production, including the boundary articulations and exclusions *that are* marked by those *practices* in the enactment of *a causal* structure” (2007: 178, italics in the original).

From this perspective, we assume that humanity can be embodied through the intra-activity of facial artifacts where the grafting of thoughts, enunciations and artifacts makes up the identity. The concept of identity we are dealing with is evidently not essentialist; it does not designate the stable core of the *Self.* Rather, we refer to identity in terms of a position-related production resulting from complex and multilayered strategies and enunciations. In line with the perspectives that have marked Cultural and Memory Studies (Foucault 1982; Kellner 1992; Thomas 2008), along with proposals from the disciplinary fields of Post-coloniality (Anzaldúa 1987; Hall 2011) and the Semiotics of Subjectivity (Violi 1997; Bianchi, Demaria, Nerggard 2002), we deviate from the idea of identity as fixity. We consider identity as the result of socially defined differences and sets of experiences, the performance of specific enunciative practices that emerge within a mediation of modes of power and biopolitical structures that makes it not a constitution according to nature but the product of the process of marking difference and eventually exclusion.

## Towards the Expansion of Humanity

As a starting point we have assumed the existence of a dominant humanistic mereology of the face based on the eyes which bear the predominant scopic function. This recognition now allows us to take a further step towards considering the importance of the face not only as a singular and unique unit but as an assembled^2^ plural apparatus whose parts have their own agency capable of intervening in the construction of identity.

In this regard and drawing on Massimo Leone’s investigation into the phenomenology of the face—an inquiry also articulated through the etymology of the words *face* and *muzzle*—we will consider the agency of the nose-mouth assemblage in facial meaning construction, setting our focus on the lower facial mereology where the boundary between human animals and non-human animals has been located. If “human animals constitute their visages because they destitute the visages of non-human animals into muzzles” (Leone 2021: w/p), our paper is interested in recognizing some facial performances where the boundary of humanity is differently placed in value through three artifacts. These restitute a differential, expanded humanity including animality within the face. More specifically, we are interested in reflecting on the practices of inscriptions that the artifacts enact in and on the face. In this regard, we acknowledge the assumption that the mouth-nose assemblage expands the human domain because it is responsible for biological life as, if organisms can survive without eyes, they are not able to do so when deprived of fundamental functions such as breathing and eating. Looking for the significant interaction that can be performed on the boundary between humanity and animality, we find three artifacts that magnify the agentivity of the mouth-nose assemblage. Therefore, in what follows, we will focus on them as they interact with the lower mereology, modifying the phenomenology of the face and performing an expansion of identity. This performance is a figurative manipulation where the eyes have been disregarded (“wasted”) and the lower face magnified. The three artifacts embody three differential facial writings (Barthes 1992) on socially perceived or self-perceived subversive forms of life, which are readable in the aesthetics of trap music, in ritualized BDSM practices and in gatherings of rave communities.

### The Subversion of *Grillz*, Ball Gags and Gas Masks

In our hypothesis it is through clothing with artifacts the lower mereology of the face that the corresponding organs can be comprehended as reconfiguring different forms of identity normative subversions. By speaking of subversion, we can recognize a semantic area linked to overturning, to sending above what was below, to spatiality. From a semiotic perspective, spatial signification is a fundamental category both for the perception of the body and for the experience of the reality that surrounds us. As far as subversion is concerned, it will also be necessary to establish a point from which to observe the overturning in question. These initial attempts at approaching the object are at the center of our reflection: is it possible to interact with the spatiality of the face and subvert the identity through artifacts?

Before answering this question, we would like to make our own the words of Judith Butler, who in *Gender Trouble: Feminism and the Subversion of Identity* (1990) writes:

“I am not interested in delivering judgments on what distinguishes the subversive from the unsubversive. Not only do I believe that such judgments cannot be made out of context, but that they cannot be made in ways that endure through time (‘contexts’ are themselves posited unities that undergo temporal change and expose their essential disunity). Just as metaphors lose their metaphoricity as they congeal through time into concepts, so subversive performances always run the risk of becoming deadening cliches through their repetition and, most importantly, through their repetition within commodity culture where “subversion” carries market value. The effort to name the criterion for subversiveness will always fail, and ought to. So, what is at stake in using the term at all?” ([1990] 1999: XXI).

Acknowledging Butler’s concerns means reversing the meaning of subversion from phenomenology to specification: what interests us is the embodiment of subversion in the face, that is, the face of those who define themselves as subverting an order or normativity that is sometimes political, economic, social, artistic, sexual. This declination of subversion, let’s say in the genitive case, allows us to highlight the possibility of an expansion of humanity displayed both within a value system, on the part of those who would like to subvert it, and in the sphere of the anti-system, on the part of those who would like to defend it. But there is something else: the face belonging to forms of life that are bearers of discourses defined or self-defined as subversive. What interests us is the artifactual dimension of subversion, or rather the subverting artifacting of identity. This dimension is dependent on the magnification of the lower face that is functional for the achievement of specific expanded properties and according to certain subversive objectives. The artifacting of identity is an augmented interfacing, an element of separation and connection between body spaces. With this in mind, three mouth-nose accessories have aroused our attention: the grill, the ball gag and the gas mask.

Despite their obvious differences in terms of artifact gestalt, functions and use, these three objects intervene in the inscription of a threshold between identity performances that contribute to questioning the normative communicative project of the face. Our approach is that by wearing artifacts such as gas masks, *grillz* and ball gags which profoundly interact with the face, the bearers expand identity performance towards a subvertive -propriation, understood as the experience of an identity performance of self-possessing for:

- Afro-American and Latin rap, hip-hop and trap artists who ostentatiously exhibit *grillz* expanding their mouths so as to *ap-propriate* the traditional class and wealth system from which they have been excluded;
- BDSM 3 practitioners who use ball gags in agreed sexual subalternation experiences expanding, that is *trans-propriating*, their wills and desires;
- The gas masks on the faces of different wearers (including police officers, protesters, steampunks and cybergoth ravers) performing an expansion between conflictive identities. While part of the equipment of the law enforcement agencies, these can be dislocated on the faces of the protesters who aim to subvert the established order. They can also be worn as a carnivalesque vestment, forming part of the disguise of steampunk and cybergoth communities that, in the context of illegal raves, *ex-propriate* the time and space of capitalist production.

With these corpora we aim to display the artifacting of different kinds of subversions. To achieve this objective, we will attempt to follow a path within the face that starts from inside the oral cavity where the grill is worn; subsequently moving to the progressive exteriority of the ball gag that protrudes from the mouth through the straps fastened around the head; up to the exterior projection operated by the gas mask that with its filters extends beyond the facial anatomy. Along this path, each artifact will be shown in its operation of expanding the mouths and noses of self-perceived or hetero-perceived subversive wearers. For each context of wearability, the paper will focus on the strategies through which the accessorized face of the bearer expresses a dissent towards a correspondent form of established (re)productivity system: in turn, class, sexuality, order and production.

If similar forms of identity-shifting performative actions could be also attributed to other artifacts such as piercings and masks, what the grill, ball gag and gas mask have in common is the technological intervention in terms of subversion with a specific organic and vital function expressed by each of them. The grill is a removable prosthesis that magnifies the biting and chewing function of the mouth, the ball gag occludes and subordinates phonation, and the gas mask is an interface that protects (or simulates doing so) the respiratory system. Along with the artifact analysis we will maintain that the interaction between the vital organ and its function and the artifact unmasks a form of identity shift. In other words, by wearing *grillz*, ball gags, and gas masks there is an expansion of the face towards a subversive figuration.

### The Mouth Endowed with a Face. Background for Imagining an Expansion of Humanity

If we accept the assumption that identity is the result of a complex communicative project also embedded in the face, then it should not be difficult to recognize it as a hyper-codified body part and to discern in its study a cross-disciplinary approach. The face is, in fact, a fundamental device for:

- recognition and identification (Gramigna, Voto 2020)—consider the perceptual phenomenon known as pareidolia (Margolis 1987; Le Grand 2001; Takahashi, Watanabe 2013; Palmer, Clifford 2020; Stano 2021) and the existence of an area of the brain dedicated to face recognition, the Fusiform Face Area (Kanwisher, McDermott, Chun 1997; Gauthier 2000; Kanwisher, Yovel 2006);
- interpersonal interaction (Buber 2020, Lévinas 1961, Goffamn 1967, Belting 2017, Todorov 2017);
- socio-cultural reading systems—as attested by the secular diffusion of physiognomy across different cultures (Evans 1969; Graham 1979; Rodler 2000; Swain 2007) and the contemporary updating of what we might call algorithmic physiognomy, i.e. the development and implementation of facial recognition systems aimed at predicting soft biometric traits such as emotions (Zhang et al. 2019) and sexual (Kosinski & Wang 2018) or political (Kosinski 2018) orientations.

Taking this theoretical perspective as a horizon, in the following sections the hypothesis that we intend to support through the analysis of these three artifacts is that they articulate an expanded discourse on corporeity, embodying an agency capable of respectively *ap-propriating, trans-propriating* and *ex-propriating* humanity towards the subversion of the identity of the bearers. By interacting on mastication, phonation and respiration, the three artifacts augment the figuration of humanity through the plasticity of meaning effects such as substitution, integration and superposition.

While approaching each artifact, we will adopt the analytical-descriptive lens of semiotics for grasping the plastic and figurative dimension of the performative shift. In addition, each artifact will be framed in its cultural context(s), by borrowing from different sociocultural theories.

The analysis will shed light on the plastic and morphologic descriptions of the artifact and its interaction with the reference organ (mouth and/or nose) and offer a brief contextualization of a few artifact uses and use resemantizations that are functional to visualizing the bearer identity shift.

**Fig. 1:**
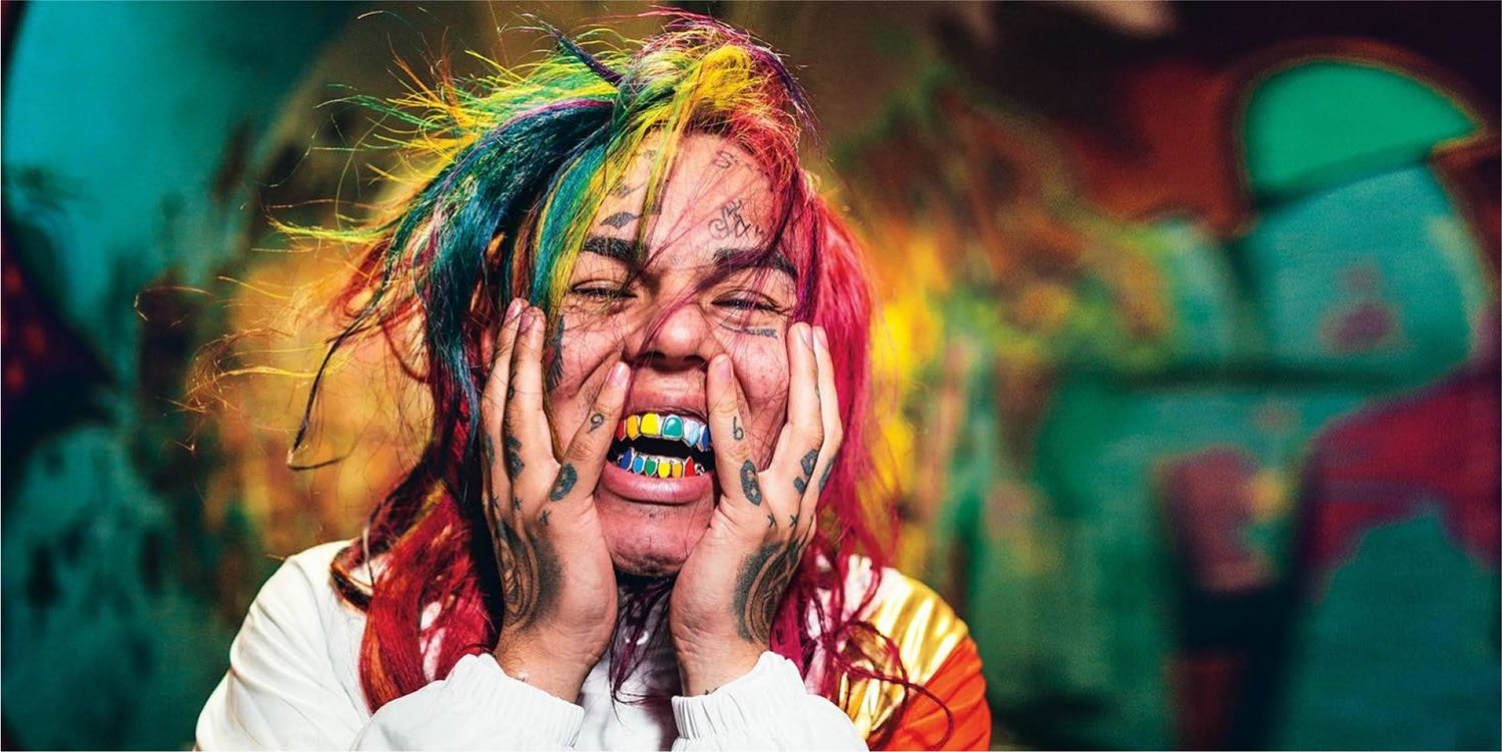
Tekashi *6ix9ine*’s rainbow mouth

**Fig. 2:**
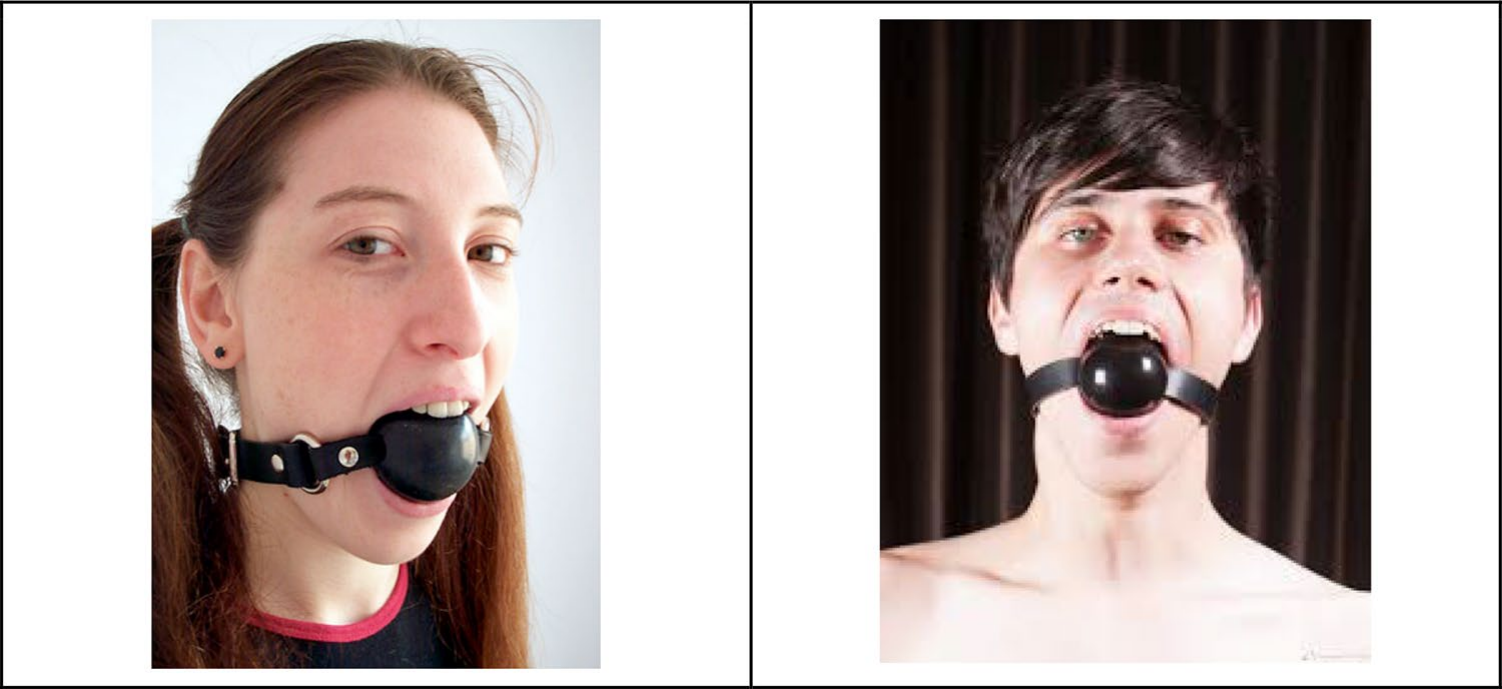
Ball gags

**Fig. 3:**
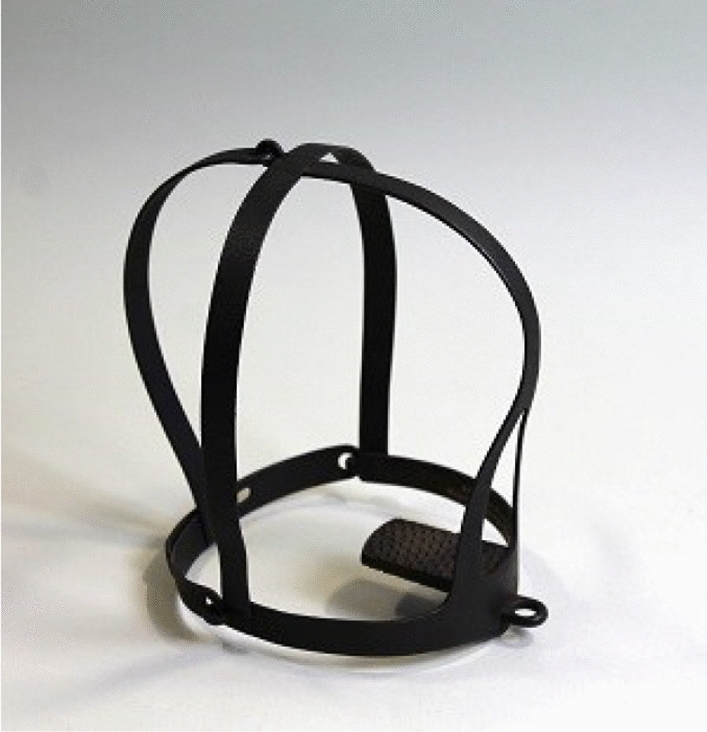
*Scold’s bridle*, Leeds Museum, 16th Century

It is worth noting that for each accessory, the study proposes a non-systematic collection of images from media or from the internet: musical, artistic and literary pieces. The heterogeneity of the corpora aims to contribute to the debate about the facial space as an unstable space of meaning construction (see Figs. 1, 2, 3).

## *Grillz* in the Mouth with Diamonds

**Fig. 4:**
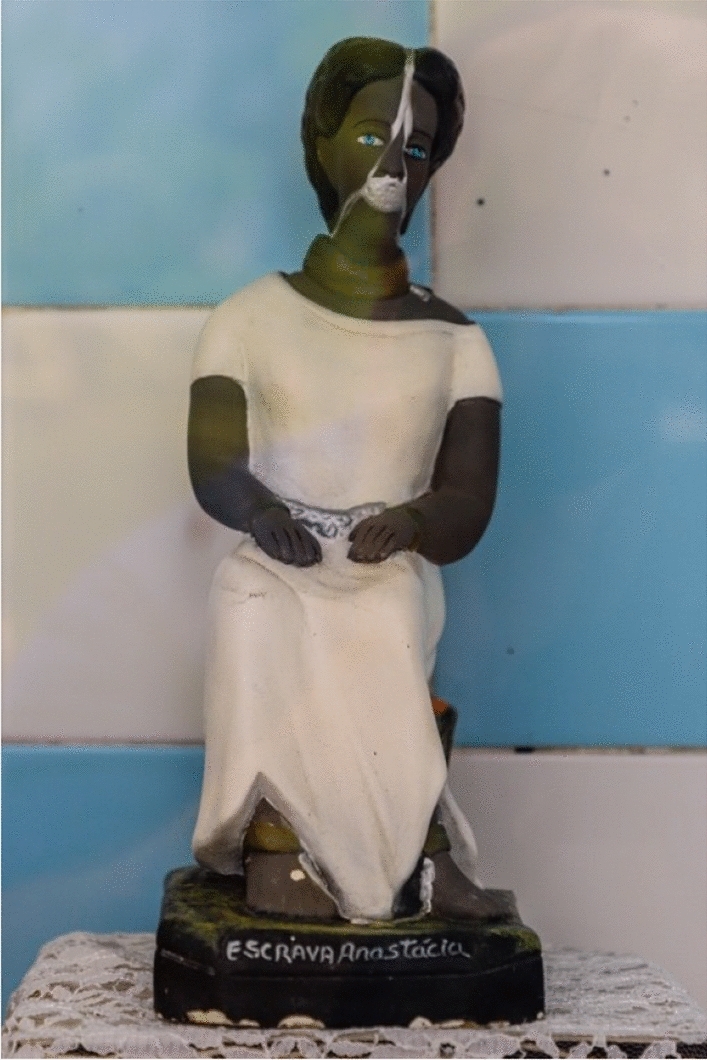
A votive statue of *Escrava Anastácia* in the *Church of the Third Order of Our Lady of the Rosary of the Black People*, Salvador, Bahia, Brazil

**Fig. 5:**
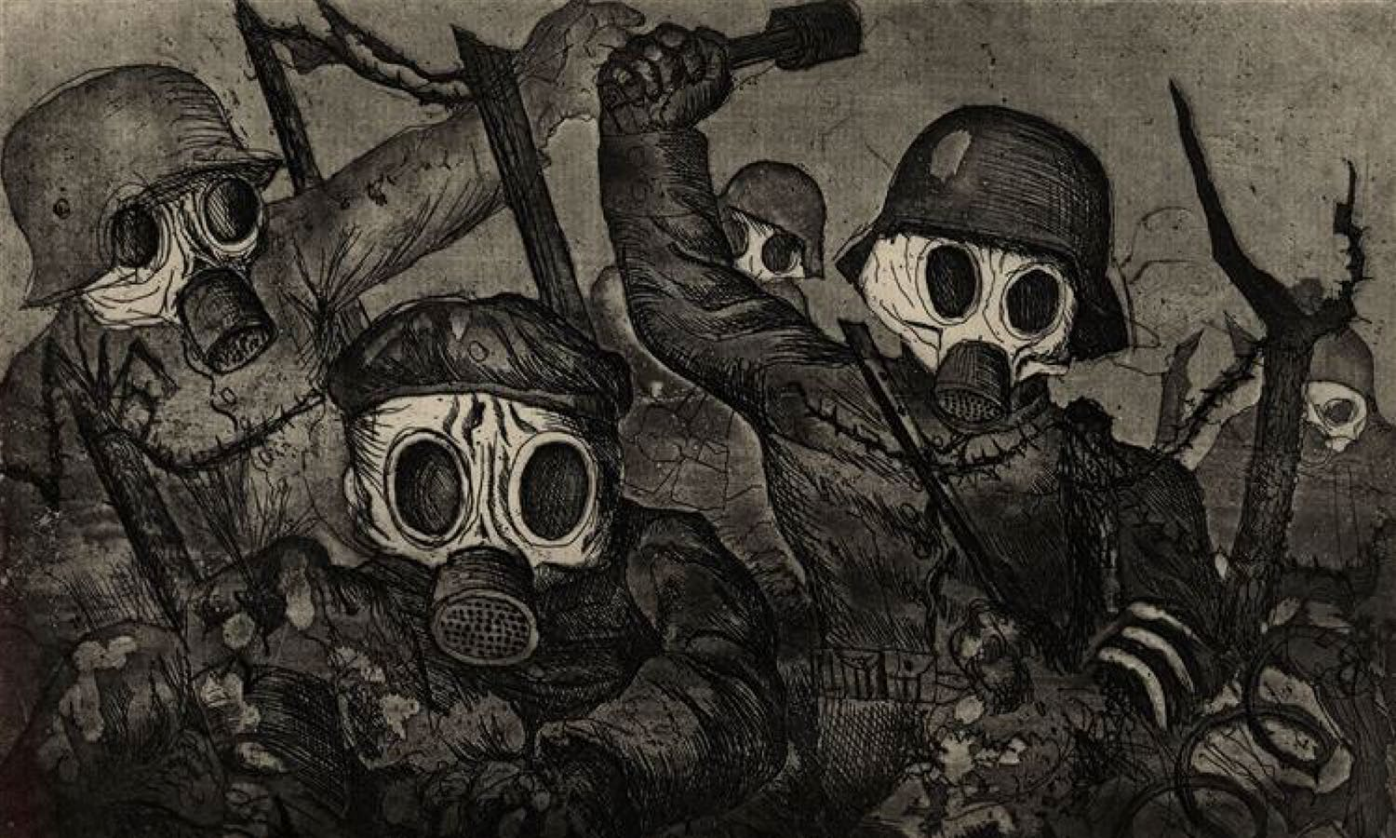
*Shock Troops Advance under Gas*, Otto Dix, 1924

**Fig. 6:**
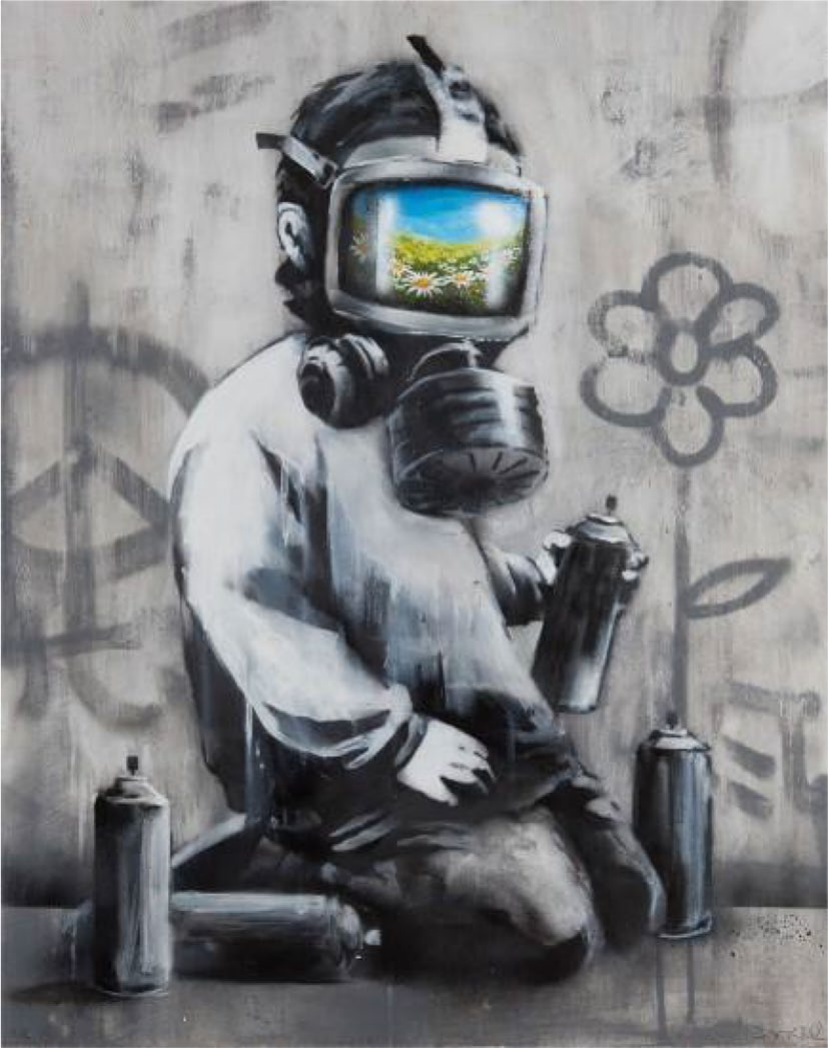
*Gas Mask Boy*, Banksy, 2009

**Fig. 7:**
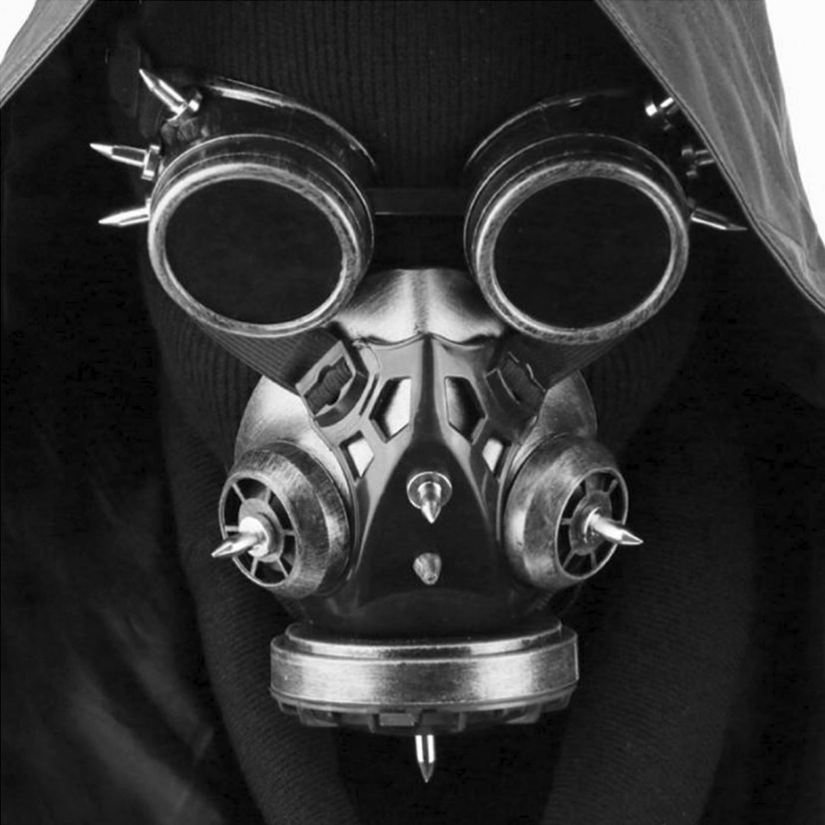
Steampunk mask

The *grill* is an artifact form of dental jewelry, made of several types of materials, among them silver, gold, platinum or precious stones. Examined from the point of view of sign production (Eco 1975), a grill derives from a dental impression, which is a negative imprint of the teeth from which a positive reproduction has been formed. Therefore, the grill is isomorphic to its imprinter, i.e., the dentition or part of it, as it is configured as an imprint, a cast that is then manipulated with expressive substances different from those of the dentition, namely, the different materials of the plating. Unlike resin-based dental prostheses, which are also made from a cast of the teeth, but are to remain concealed and *look real*, *grillz* expand the teeth with different colors and textures (e.g., golden or even rainbow-colored *grillz*—as in the mouth of the artist 6ix9ine; Fig. 4) and by doing so they superpose on the oral cavity a fake-looking reproduction. Through such superposition, we hypothesize that wearers of *grillz*, such as Afro-American and Latin rap, hip-hop and trap artists, place within their oral cavity the image of wealth and prosperity (see Figures 5, 6, 7).

Indeed, if within orthodontic therapeutic principles devices are intended to replace the function of missing or defective teeth, *grillz* do not perform any functional purpose but only an aesthetic one. The chromatics of the gold and diamonds that often characterize this ornament create a point of light on the oral cavity that arguably contributes to the loss of the hypnotic primacy of the eyes and even the lips within the facial configuration. The inside of the mouth is thus magnified, and so are functions such as biting and chewing (whose organs are covered in flashy materials).

The artifact started to be worn in the 80 s by hip-hop and rap artists mainly from the South of the United States such as Raheem the Dream and Kilo Ali (both from Georgia). From the cultural and geographical periphery of the United States, *grillz* became progressively popular among New York artists thanks to an immigrant of Surinamese descent, Eddie Plein, owner of Eddie's Gold Teeth, who is frequently credited with having brought the trend to the center of the music semiosphere by starting to make these devices in his Brooklyn basement. The trajectory of *grillz* can be traced in a variety of textualities diffused on the Internet, social media accounts of artists who show off their golden or diamond mouths or even songs dedicated to the artifact such as *Grillz*, by the American rapper Nelly, an anthem in honor of gold- and diamond-studded teeth.

Thanks to this recent increase in the *grillz*’ popularity, they gradually started to adorn the teeth of mainstream pop artists such as Katy Perry, Madonna, Miley Cyrus, and Beyoncé and eventually even appeared in Fashion Weeks. This movement from the periphery to the center of the cultural semiosphere contributed to leaving behind the artifact’s initial supposedly disturbing function and turning it into a jewel for the mouth.

Regarding the flashy dimension of the grill, which also contributes to the above-mentioned anti-mimetic effect, it can be framed within the *bling bling aesthetic* (synesthetic onomatopoeia reproducing the sound of jewelry). The term *bling* according to the *Oxford English Dictionary* “represents the visual effect of light being reflected on precious stones and metals.” Stemming from rap vernacular, the term has recently been more generally appropriated by the language of popular culture. In the homonymous track Bling-Bling recorded by a group known as the Cash Money Millionaires, *bling bling* stands for a container of ostentatious displays of wealth, embodied by a series of accessories, among which are *grillz*:

Medallion iced up/Rolex bezelled up d my pinky ring is platinum plus/Earrings be trillion cut/

And my grill be slugged up/My heart filled with anger ‘cause nigga I don't give a fuck/

The marked visibility of these accessories in Afro-Caribbean, Afro-American and Latin musical cultures, which have given rise to genres or artistic fields such as rap, hip hop, reggaeton and trap, arguably points to the thematization of a subversion of status or to what the researchers Alf Rehn and David Sköld (2005) called the trope of re-appropriation in the *bling bling* performance of capitalism.

The conspicuous consumption habits exposed by *bling bling* fashion can be related to an attempt in the late twentieth century and early twenty-first century US context to substitute the socio-economic conditions of Afro-Caribbean people and Afro-Americans by appropriating the images of wealth. As art historian Krista Thompson [2009] points out:

“While some rappers in the past had shone a critical light on capitalism, hip-hop artists in the post-soul period unabashedly celebrated materialism or a ‘guerilla capitalism’, draping themselves in symbols of wealth, from gold chains and medallions to all manner of brand-name goods.” (2009: 483).

Away from the urban and artistic scenes of the above-mentioned genres, precious materials such as gold have been used in dentistry to fill cavities (so-called ‘crowns’) and to make dental restorations. However, unlike the grill, which is removable, gold in the mouth for dental use is properly encapsulated, and therefore fixed.

This removable dimension of the grill, which articulates an iterative expectation—the accessory can be put on and taken off as needed –, as opposed to the durable dimension of the dental prosthesis, highlights the non-fixed property of its support, namely, the teeth, the mouth and the face as a whole. In this face cavity—the mouth—is written the history of actions in progress, a substitution that subverts the expectations of a social class and can be denied.

## Gagging Oral Pleasure

The ball gag is an artifact used in BDSM role play and its main function is to fill the mouth; by so doing the ball gag expands the submissive role during the sexual experience. The concept BDSM normally refers to a wide range of sexual practices involving bondage, discipline, domination and submission, sadism and masochism. This definitory term embraces a variety of ways of experimenting forms of eroticism and sexuality focused on highly codified role plays. The acronym works as an isotopy which includes a range of preferences that relate sexuality with physical and mental pleasure, control and pain. A common thread within BDSM practices, as they are organized around the performance of different roles, is the construction of a ritualistic link between eroticism, pain and bodily stimulation. In BDSM practices, the perception of pain is turned into a subjective expression and a consensual choice, and ultimately a point of contact between the sensible and the intelligible dimensions of identity while it is plurally managed. In this vein, BDSM principles stem from the search for a deeper comprehension of pain that can be co-experienced and therefore coped with, integrated by the presence of the other. According to this vision, the vulnerability of the body can be felt through a mutual management of consent that also passes through the use of facial artifacts.

Within BDSM, by wearing the ball gag it is possible to consensually expand the inscription of the dominant, who *stricto *sensu regulates the subalternate possibility of speech, in the face of the submissive, who cannot speak. This artifact, which emerges from the mouth and is fastened around the head by means of straps, during performances can subvert the sexual dimension of identity.

One of the most remarkable propositions upon which BDSM relies is the perception of the body as a substrate or rather: “a process of materialization that stabilizes over time to produce the effect of boundary, fixity, and surface we call matter” (Butler, 1993: 9). This progressive materialization: “is also directly involved in a political field; power relations have an immediate hold upon it; they invest it, mark it, train it, torture it, force it to carry out tasks, to perform ceremonies, to emit signs” (Foucault [1976] 1995: 25). From a semiotic point of view, we can refer to the process of materialization of bodies as “lines of resistance and possibilities of flow, as in the grain of wood or marble, which make it easier to cut in one direction than in another” (Eco [1997] 2000: 39).

By considering such perspectives, the body would present an ontological base with its own constitutive morphology that would subsequently be negotiated by the practices and discourses that shape, regulate and model it. Within BDSM practices, the process of materialization responsible for producing the effect of boundary of the bodies and the relationships among them is primarily regulated by a dimension of subjective and consensual subordination, which is expressed through various practices of obstruction: of mobility, of vision and phonation, among others.

Let us look at an exemplification from visual culture: in his photographic series *Gay Semiotics* (1977–1979), a visual encyclopedia of the forms and lifestyles of gay culture at the time engaged in a widespread campaign to gain social visibility in the city of San Francisco (Voto 2020), Hal Fischer puts the ball gag—depicted as a gay mask—at the center of this figurative scenario. The photographic series sheds light on the practices, artifacts and discourses with which homosexual communities brought out a discursive materialization of masculinity outside of heteronormativity. We read in the portrait dedicated to the ball gag:

“The gag mask is constructed of a black leather strap and a two-inch interior gag which is contained in the wearer’s mouth. The gag mask has two basic functions. First, it provides the wearer with oral stimulation while his partner is involved in other activities. Second, it keeps vocal participants quiet, an important consideration for S[ado] and M[asochism] apartment dwellers.” (https://www.gaysemiotics.com/gay-semiotics).

In Fischer’s description, the ball gag is codified through two functions: stimulation and occlusion. This double agency of the artifact, designed at the same time for pleasure and pain, is not just a feature of BDSM imaginaries. Facial artifacts for stimulation and occlusion, for the integration of the other in the mouth, can also be found in contexts where submission is not a consensual choice but a violent constriction. The fundamental and situated distance between BDSM and these other settings is what is represented by non-consensual subalternation and patriarchal violence incarnated in other facial artifacts arising from the Medieval Inquisition and subsequently transferred to colonial exploitation, such as the Scold's bridle or the *mask of speechlessness* (Kilomba 2021). In this sense it is of great interest to reflect on the material meanings that the shift to consensuality inscribes in BDSM practices, for if the Scold’s bridle or mask of speechlessness was mainly composed of metal—a materiality which hurt and restrained the wearer—the ball gag finds in leather and plastic its differential affordance capable of subverting sexual identity. The Scold's bridle, also known as the gossip’s bridle^4^ (Federici 2012), was: “a metal contraption also used to muzzle slaves that enclosed the wearer’s head and, if she attempted to speak, lacerated her tongue. Gender-specific forms of violence were also perpetrated on the American plantations” (2018: 14). Within this perspective it is worth mentioning the popular folk saint *Escrava Anastácia*—Anastasia the Enslaved—venerated within the Umbanda tradition^5^ and depicted as a slave woman of African descent, always wearing the speechlessness mask and endowed with extraordinary beauty. As affirmed by Grada Kilomba in her *Plantation memories. Episodes of everyday racism,* this facial artifact can be said to: “represent colonialism as a whole. It symbolizes the sadistic politics and its cruel regimes of silencing the so-called ‘Others’” (2010: 16).

Both artifacts, the ball gag and the mask of speechlessness, are designed to expand the subjectivity of the wearer into subordinate constraint. Nonetheless, in the case of the mask of speechlessness we witness an artifact of subjugation and exercise of violence and power, whilst in the case of the ball gag we can talk in terms of trans-propriation of subalternity towards a differential conception of humanity by performing a subversive form of sexual submission. In this latter case, thanks to the consensual negotiation of vulnerability, the spatiality of the face is plurally managed and thus expanded. Arguably, by wearing a ball gag the individual bearer voluntarily and consensually occludes phonation in order to transfer the possibility of speech and enunciation consensually and intersubjectively, or perhaps we could say trans-subjectively.

## The Gas Mask: *Low* and Disorder

The gas mask is a facial artifact consisting of a tight-fitting facepiece that contains filters, an exhalation valve, and transparent eyepieces. From a functional point of view, it is primarily a breathing device: by filtering and cleaning the air, the mask protects the user from inhaling airborne pollutants and toxic gasses. Specialized studies have further defined the gas mask as an “interface that combines inspiratory and expiratory resistances, and that has an instrumental dead space, which possibly results in a rebreathing phenomenon” (Bourassa et al. 2018:1).

The gas mask needs to be superimposed on the facial organ, in order to perform its function as a respirator. Such interaction therefore creates an additional layer that augments the facial anatomy. In fact, the filter cartridge near the mouth either directly, or via a flexible hose, protrudes from the face, projecting it towards its exteriority, as do the drinking tubes which may be connected to a water bottle.

At the same time the respirator masks the face, forming a seal over the nose and mouth that covers them and also obscures the eyes and other vulnerable soft tissues. In this study perspective, by rendering the wearer’s face unrecognizable and indistinguishable the gas mask marks the performance of a turning of individuality into collectivity.

In the following paragraphs, this will be demonstrated by means of a brief review of collective (and conflictive) uses of gas masks in recent history.

Mass-produced gas masks started to be commercialized during the First World War, in correspondence to the extensive use of chemical weapons. In particular, this kind of mask was introduced by the Allies in response to the asphyxiant gases used by the German troops. In Otto Dix’ *Shock Troops Advance under Gas*, this artifact exacerbated the terrifying ghost-like semblance of soldiers’ faces that epitomized the dehumanizing effect of the war.

In concomitance with the further sophistication of the war industry after the Second World War and the introduction of nuclear and biological weapons, the filtering system of the gas masks became more and more accurate and universally identified by a code of colors. During the Cold War, the permanent threat of a nuclear attack turned the gas mask into daily life equipment. In the USSR, the Soviet population was issued with the GP-5 gas mask whose production started in 1962 and ended in 1990.

Besides the specific war context and nuclear menace, the gas mask is part of the security and protection devices with which specialized professions such as hazmat workers, firefighters, and doctors in different fields are provided. Law enforcement is another field that includes the gas mask in the workforce’s equipment, to be used mostly in case of civil unrest. Unrest and riots are the situations where security forces are allowed to use substances such as CS gas (2-chlorobenzylidene malononitrile), also known as “tear gas”, for crowd control against civilians. On such occasions, according to law enforcement guidelines the gas mask should protect the police force from the incapacitating effects of exposure to tear gas, such as coughing, shortness of breath, and a burning sensation in the eyes, mouth and nose. Recalling the observations about gas masks augmenting plasticity, we can maintain that by extending the police officers’ anatomies, the gas mask effectuates visual intimidation. Moreover, by resembling the war imagery above mentioned, gas masks appear to be part of dehumanizing military gear.

Curiously, if one looks at the contemporary iconosphere, the gas mask has come to represent, more than the order represented by the police, the counter-forces opposing such order.

Indeed, this device has been widely featured in murals, stencils and other street art expressions, for instance, in the popular Banksy *Gas Mask Boy*, portraying a figure whose respirator, in a visual paradox, reflects a blooming field.

Seemingly, in blogs and handbooks aimed at preparing people involved in acts of civil disobedience, protesters are trained to build DIY gas masks: *Homemade Gas*, exhibited at the Museum of the City of New York in 2015, showed an assortment of pieces that demonstrate the various techniques used by activists to make facsimiles of gas masks. On the protesters’ side, this artifact helps make it more difficult for police and surveillance systems to identify the protesters’ identity. It is worth mentioning the double protection function (from the virus, in this case, and from the surveillance system) that, similarly, the sanitary mask performed during the recent protests against police brutality towards Afro-Americans, sparked by the killing of George Floyd in the US. This transferability of the functions embedded in the gas-mask assemblage evidences at the same time the wearable nature of the wearers’ subjectivities.

### From Riots to Rave

The anthropologist Didier Fassin, in his ethnography of the police in France (2013) and in particular the *Brigade Anti-Criminalité* (la BAC), an elite unit of the national police, reflected on how these bodies attempt to subjugate the ZUS, the *zones urbanines sensibles*, where all kinds of illegal activities are said to be harbored. In the analysis made by the French anthropologist of the force of order discourse, the French banlieues would therefore represent a blot on the urban landscape, temporally removed from order and which must be returned to it. In this regard, the ZUS can be considered a form of TAZ or *Temporary Autonomous Zone*, as theorized by the American anarchist Hakim Bey [1991], an area where the established order is temporarily suspended.

Bey presented the concept of TAZ in a long elaboration in his work *TAZ: The Temporary Autonomous Zone, Ontological Anarchy, Poetic Terrorism,* focusing on the socio-political tactic of creating temporary spaces that elude formal structures of control. Among other characteristics, notably, the insurrection envisioned by Bey has a festive character: “The uprising is like a saturnalia which has slipped loose (or been forced to vanish) from its intercalary interval and is now at liberty to pop up anywhere or when. Freed of time and place, it nevertheless possesses a nose for the ripeness of events, and an affinity for the genius loci” (1991: 103).

By following this festive dimension of insurrection vaticinated by Bey, the TAZ became “a major rallying cry for the embryonic rave culture in England” (Sellars 2010: 89), which borrowed from Bey in its nascent scene the narrative of liberation of space and consciousness operated by techno music. In their description of the warehouse party scene in Blackburn, Ingham and Purvis identified a clear resonance with Bey’s TAZ:

“It was the temporary and transgressive nature of the warehouse parties that made them special, feelings amplified by the illegal use of the premises and often by the consumption of drugs. There was a sense of adventure in the exploration (or creation) of a territory at once known to the majority of partygoers, who were in large part local inhabitants, yet also excitingly unfamiliar in its newly revealed potential for transformation through sound” (1999: 294).

The unfamiliarity of the partygoers is also connected with the fact that they were the bearers of new aesthetic settings characterized by very flamboyant and diverse rave fashions which, nonetheless, acquired common elements at international level. In this vein, gas masks appeared among other accessories, such as safety vests and protective suits, as part of the ravers’ customary clothes. In particular, subcultures such as steampunk and cybergoth that combine rave fashion with respectively goth and futuristic elements have adopted the gas mask as a distinctive feature of their styles.

Refunctionalized, as part of a carnevalesque costume, the gas mask marks a reversal ritual that places the disguised subjectivities in a kind of separated locus where norms are suspended.

Dislocated in the context of the rave, the gas mask is denuded of its respirator function that becomes a purely a-functional and ludic simulation.

By turning into a costume, it therefore performs a collective ex-propriative ritual: the urban places traditionally related to Fordist production such as factories and warehouses “change (…) their meanings within a rapid and not painless transformation of uses and purpose” (Ilardi 1990). In fact, the ravers expropriate the *terrain vague*, the industrial void that, through the party, is given a new recreational function. Such new meaning, antithetic to capitalist production, arguably operates a time expropriation as well. In this regard, our hypothesis is that during the rave, the techno music, through its sampling operation, expropriates the *tempo* of the music and transforms it into *beats per minute* (BPM). Similarly, the cybergoth or steampunk accessories articulate an oxymoronic combination of times: the past in the case of goth and steam subcultures, the future in the case of cybers.

When the gas mask is detached from its vital and organic function and turned into a pure accessory, the conversion of this object into a sign of its use, by recalling the Barthesian reflection, seems to be complete, as does the identity of the artifact bearer when he/she simulates a nuclear apocalypse while dancing.

## Final Thoughts

The present work, stemming from a preliminary study of the authors, has been undertaken to investigate more deeply a fairly unexplored territory beyond the hyperbolic ocular isotopy that rules the aesthetics and the semiotics of face representations so as to comprehend the meaningful potential of other facial organs, in this case the mouth and partly the nose. If faciality, at least under the Western representational frame, has been overwhelmed by its interdependence with the eyes, as resonates in the refrain of the Billy Idol song—“such a human waste your eyes without a face”—the present work has tried to focus on the lower facial mereology. In the authors´ vision, this still unexplored spatiality of human faces is where the expansion of humanity towards plastic and figurative features of animality can be grasped. Through the present study we believe that we have contributed to enriching the debate around the unstable meaning of the human face, focusing on the agency of facial artifacts in operating such meaning displacement.

The reflections and findings from this study need to be interpreted in the light of several limitations. It is important to recognize that the corpora analyzed in this work tend to a certain heterogeneity, by mixing together works of art and music, pieces of literature, visual and textual discourses circulating in the Web. The heterogeneity of the corpora is not aimed at systematically problematizing the artifacts within a certain (sub) cultural context embodied by the artifact bearers as a population. Rather, the variety of examples is designed to contribute to the debate about the facial space as an unstable space of meaning construction where different agencies negotiate facial spaces and times and reconfigure entanglements among them. Also, the literature review does not purport to construct a solid disciplinary framework. Rather, this study has opted for diffuse cross-contamination among disciplinary fields that have functionally served the analysis, without any pretension of exhaustivity in any of them.

For a greater effort at systematizing, future researchers may wish to study the phenomena examined here using more homogeneous corpora, in order to gain more depth into the single accessories and their corresponding cultural backgrounds.

## Footnotes

1 The intertextual recurrence of this hyperbolic ocular isotopy has also been contemplated by Billy Idol himself who, in his autobiography *Dancing with myself*, explains how he “was particularly intrigued by the ‘60 s French nouvelle vague forerunner *Les yeux sans visage*, about a brilliant plastic surgeon who vows to restore the face of his daughter, who has been horribly disfigured in a car accident” (2014 w/p). The film is the adaptation of the noir novel *Les yeux sans visage* by French writer Jean Redon, published just one year before Georges Franju’s movie (1959).

2 The concept of assemblage, which from Gilles Deleuze and Félix Guattari’s proposal (1890) is redeveloped by Donna Haraway (1985), Bruno Latour (2005) and Manuel De Landa (2016), refers to the network of relationships that runs through not only the biological world but every material entity, living and non-living, human and non-human, defined by its own relationships that are thought of as necessary.

3 As will be explained in §6, BDSM is an acronym that stands for bondage, dominance and sadomasochism.

4 In her *Witches, Witch Hunting and Women* Silvia Federici analyzes the semantic overturning of the word ‘gossip’, tracing the shift from “its positive connotation of female friendship to the negative one that refers to malignant speech, alongside the parallel degradation of the social position of women spearheaded by the witch hunt” (2018: 2).

5 Umbanda is a syncretic religion that has blended African traditions with Catholicism, Spiritism, and Native American beliefs and has spread across Brazil, Argentina and Uruguay.
